# Directional Water Wicking on a Metal Surface Patterned by Microchannels

**DOI:** 10.3390/ma14030490

**Published:** 2021-01-20

**Authors:** Nima Abbaspour, Philippe Beltrame, Marie-Christine Néel, Volker P. Schulz

**Affiliations:** 1 UMR1114 EMMAH INRAE—Avignon Université, F-84914 Avignon, France; marie-christine.neel@univ-avignon.fr; 2 Department of Mechanical Engineering, Baden-Württemberg Cooperative State University Mannheim, Coblitzallee 1-9, D-68163 Mannheim, Germany; volker.schulz@dhbw-mannheim.de

**Keywords:** directional wicking, patterned surface, wetting dynamics, capillarity, 3D simulation, Volume of Fluid, Lattice Boltzmann Method, Selective Laser Melting Manufacturing, microstructure

## Abstract

This work focuses on the simulation and experimental study of directional wicking of water on a surface structured by open microchannels. Stainless steel was chosen as the material for the structure motivated by industrial applications as fuel cells. Inspired by nature and literature, we designed a fin type structure. Using Selective Laser Melting (SLM) the fin type structure was manufactured additively with a resolution down to about 30 μm. The geometry was manufactured with three different scalings and both the experiments and the simulation show that the efficiency of the water transport depends on dimensionless numbers such as Reynolds and Capillary numbers. Full 3D numerical simulations of the multiphase Navier-Stokes equations using Volume of Fluid (VOF) and Lattice-Boltzmann (LBM) methods reproduce qualitatively the experimental results and provide new insight into the details of dynamics at small space and time scales. The influence of the static contact angle on the directional wicking was also studied. The simulation enabled estimation of the contact angle threshold beyond which transport vanishes in addition to the optimal contact angle for transport.

## 1. Introduction

In microfluidics, water forms droplets which remain pinned on solid surfaces. The transport or spread of these droplets is a challenging task in an industrial framework as lubrication, chemical reaction, heat exchanger, and water management in fuel cells. In most microfluidic devices, liquid motion is provided by a pump or an external energy input such as piezo elements. Nevertheless, surface heterogeneity as a gradient of wettability, i.e., the solid-liquid contact angle, allows droplet spreading without any external energy input [1,2]. It is a relaxation dynamics of the drop/surface system which decreases its free energy. Such liquid movement is usually called *passive transport* when the liquid flow has a privileged direction. Passive water transport at microscale offers many applications [3,4].

Such a directional transport also appears in the living world. Some species such as insects, lizards and plants living in deserts use it to harvest water [5,6,7,8]. Heterogeneous wettability driven liquid motion was also observed on wharf roach skin [9], and its frequent use by living organisms inspired a variety of industrial applications [10,11,12]. In all these examples, it is the surface heterogeneity that causes liquid drops to move. Heterogeneity can result from spatial variations in the wettability [13,14,15] but can also be caused by small scale (micrometric) topographic structures on the smooth solid surface acting as an asymmetric capillary [5,6,7,16,17,18]. The water spreading through these microchannels is called water wicking in the literature [16].

The initial motivation of this work is the use of such a passive transport to remove the liquid water excess in the Proton-Exchange Membrane (PEM) fuel cell [19]. Indeed, water management is a crucial issue as evidenced by several patents and literature on this topic over the last decade. Part of researches has focused on the design of the channels containing the reactant gas flow and water in the bipolar plate [20,21,22]. Another possibility is to implement additional micro-channels to drive the water by capillarity in the bipolar plate [23] or in the membrane [24]. A third possibility is the improvement of the shape of the channel cross section [25,26]. Passive transport by modifying the surface properties of the channel is an innovative approach that has not been studied in the framework of PEM Fuel Cell (PEMFC). Transport using gradient wettability requires a physicochemical treatment applied to the metal surface [27,28,29]. The drawback of this treatment is the possible modification of the metal properties as conductivity and the durability of this coating. Thus, we turned to the water wicking due to microstructure on the surfaces as in [30].

The realisation of such structures in the PEMFC context requires that chosen materials are compatible with low temperature hydrogen fuel cell constraints, i.e., robust and electrical conduction. These constraints raise open questions that have not been addressed in the literature. Indeed, experiments having reported passive directional water motion driven by microstructures [5,6,7,9] used other liquids than water and other materials than metal. Then, there is no evidence that reproducing the pattern found in literature will lead to water wicking because water on metal is less wettable than other couples liquid/substrate used in the previous references. Moreover, if the technology progress of additive manufacturing allows complex geometry designs in metal, especially in the field of fuel cells [31,32,33], none have been made, to our knowledge, at the scale of a few tens of microns.

The main goal of the present paper is to manufacture micro-sized pattern on a metal surface in order to obtain spontaneous directional water drop wicking. It is known that open microchannels are acting as micro-capillary [34] and spreading a drop even if the liquid is poorly wettable on the substrate. The directional spread is obtained only if the structure is asymmetric. We considered open capillaries constituted by repeated fin-shaped patterns such as those proposed by [16]. Drawing on the latter reference, we present an experiment that shows the directional spreading of a water drop on the patterned surface. The experiment was prepared and interpreted by numerical simulation of the evolution of the drop. Several numerical simulations have analyzed the evolution of velocity field, water content, interfaces and pressure in the latter case [1,2,13,14,15,27,28,29,35]. Theoretical approaches to drop wicking in channels of complex geometry [16,17] merely use semi-empirical formulas in the framework of quasi-static flow. The main idea of the directional behavior lies in the hysteresis phenomenon of a front liquid arriving at an edge: a threshold of the advancing contact angle should be overcome to further advance according to the Gibb’s criterion [36]. The relevance of this criterion in our case is analysed in Section 5. However, none of these references have attempted to simulate the time evolution of liquid drops far from quasi-static regime in complex geometries such as the fin-shaped structures considered by [16] nor a periodic distribution of tilted pillars [18]. A presentation of the experiment and a first numerical analysis reported in [37] suggested that the fluid inertia cannot be neglected during the wicking. Simulating a free flow surface wetting dynamics with the complete Navier-Sotkes in a complex geometry is a challenging task. The simulation is performed using two different approaches: the Volume of Fluid method and the Lattice Boltzmann Method [38]. The advantages and the limits of each method is discussed in the paper.

The paper is organized as follows: in the next section, we specify the constraint due to the PEM fuel cell framework and the manufacturing (Section 2). Then, the results of the drop wicking experiments are reported in Section 3. After presenting the models used for the numerical simulations we analyze the simulated dynamics in Section 5. Finally, Section 6 summarises our main results and the future outlook.

## 2. Problem and Method

We applied numerical and experimental approaches to predict and evaluate spontaneous liquid displacement on manufactured structures made by micro laser sintering with stainless steel.

### 2.1. Structure Geometry

**Figure 1:**
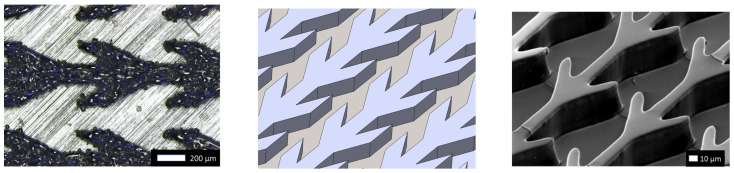
Fin shaped structures added to a flat surface. Top views of the fin-shaped structure studied here and manufactured by Selective Laser Melting (SLM) in a picture from above (**left**), CAD in an isometric view (**middle**), and original Polydimethylsiloxane (PDMS) part (**right**). Notice the different scales and compare the rough metallic structure manufactured by SLM (**left**) with the smooth surface considered in [16] made of resin (**right**).

The structure of the micro-channels is based on the fin-shaped one on which [16] observed one-directional wicking. Figure 1 represents, left panel, the micro-channels considered here and, right panel, the original version of [16]. As can be seen on Figure 1, the studied channels are about four times wider and two times deeper than the original version of [16]. At larger scale the one-directional wicking is not a priori guaranteed since the experiments conducted in [17] demonstrated that pattern scale influences spontaneous directional wicking, which [16] found to be more rapid on thinner channels.

The fuel cell framework also imposes the use of a material that significantly differs from those used in [39]. These changes are discussed below.

### 2.2. Material Constraints

A robust material that conducts electric current and resists corrosion and heat is highly desirable for our purpose. Stainless steel satisfies these requirements. Since [16] found smaller patterns more efficient, we manufacture our structures by the Selective Laser Melting (SLM) which is adapted to steel and achieves high resolution. Nevertheless, sizes as small as in [16] are out of reach. Moreover, the SLM technique cannot avoid slight deviations between CAD design and manufactured component, which we also expect to influence wicking.

Finally, the reaction product in a PEM-fuel cell is pure water, which returns a water/steel contact angle larger than in the drop transport experiment [16,39]. The latter was performed on Polymethylmethacrylate (PMMA) and Polydimethylsiloxane (PDMS) substrates, which of course do not satisfy our robustness and electric conductivity requirements. Moreover, reference [39] associated these substrates with IPA and soapy water. Since it seems that drop wicking on a given structure is decreased when the contact angle is increased, we expect to find the optimal conditions upon exploring the 0–70 degrees range. Numerical simulation is the most flexible tool to study the influence of contact angle. However, it needs to be validated by experiments. Our method consists in combining these two approaches.

### 2.3. Method

**Figure 2:**
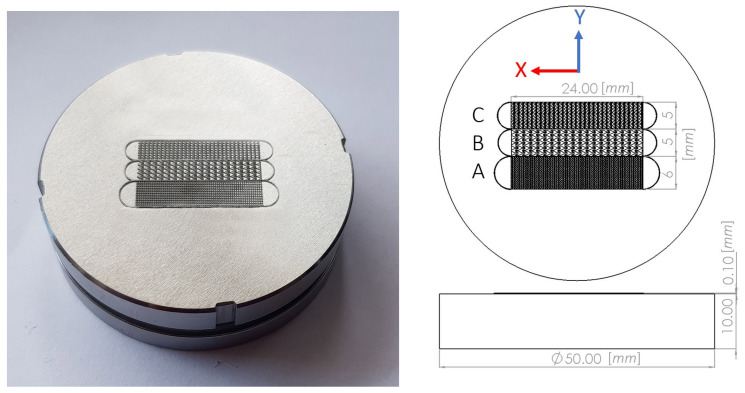
Three adjacent structures exhibiting fin shaped channels added to the steel base by SLM. (**left**) Actual A, B and C structures. (**right**) CAD design documenting the total length and width of each structure, in millimeters.

A numerical approach using either interface tracking by Volume of Fluid (VOF) or the Lattice Boltzmann Method (LBM), captures drop motion in a channel limited by the patterns described in Figure 1. We will see in Section 5 that interface tracking suggests directional drop motion at contact angles smaller than 70 degrees. The numerical approach was validated by an experimental set-up on steel structures based on the geometry introduced by [16]. Liquid transport was observed immediately after a distilled water drop had been placed on each one of three steel structures manufactured in-house and exhibiting the fin-shaped geometry of Figure 2 with slightly varied scales. It turns out that channel width influences spontaneous drop displacement. Manufacturing and measurements are detailed in Section 3. After a brief description of the underlying physical model in Section 4, numerical issues are detailed in Section 5 which also compares the simulation results with the experiment.

## 3. Experimental Study of Liquid Wicking on Fin-Shaped Patterns

The manufacturing process of adding fin-shaped patterns of various widths on the steel surface is the first step of our experiment. The next step consists in observing drop evolution on the structure thus obtained.

### 3.1. Substrate Manufacturing and Physical Properties

**Figure 3:**
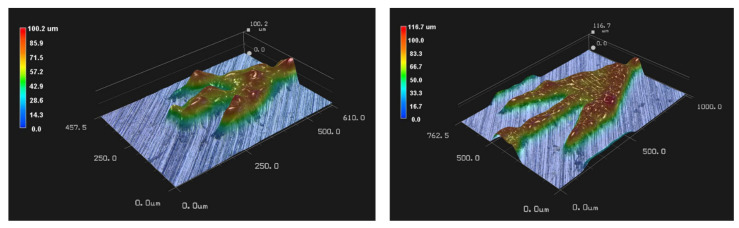
The pictures represent the basic element of structure manufactured by SLM. (**left**) Structure A and (**right**) structure C of the experimental set-up (Figure 2) corresponding to the smallest and largest lengths specified in Figure 4. Colours indicate height measured from the steel platform.

As in [16] the substrates used here to study drop wicking are flat surfaces with fin patterns forming channels. One of them is shown in Figure 1. The platform and patterns in the present study were made of 1.4112 stainless steel, and three such structures denoted A, B and C were built on a single platform (Figure 2). For manufacturing first the CAD data is provided (Figure 1 middle panel). In second step, the CAD data is converted to layers. In the third step of manufacturing by Selective Laser Melting (SLM) the machine deposits an uniform thin layer of metal powder on the surface and melts each layer successively onx the platform by a laser beam. The past two steps of spreading the uniform powder layer and sintering are repeated until the channel wall height reaches the desired value which here is 100 [μm] (shown in Figure 2 right panel). Then, the powder residue must be cleaned and the manufacturing is completed. A single cell of the geometries from structure A and B are depicted in Figure 3 left and right respectively. The height of the structure is visualized by a 3D microscopic scan. The deviation from CAD design of the structure A is relatively larger than for the C structure. For the structure A, the roughness is not negligible compared to the size of the structure.

**Figure 4:**
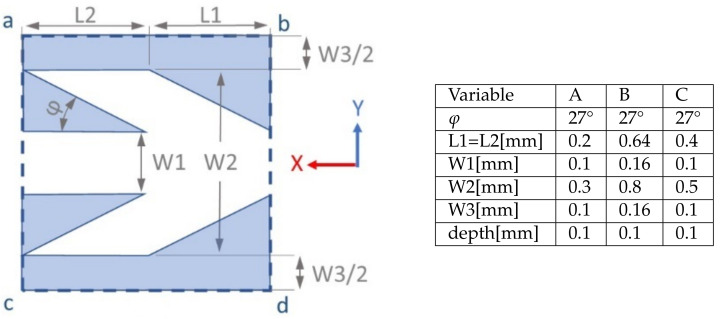
A single cell of the pattern from which is deduced the capillaries. The blue colored part is the fused melted metal with a height of 0.1 mm. The bottom of the capillary colored white. Both parts create an elementary cell which is included in the rectangle ‘abcd’. Adding copies of this rectangle adjacent to its parallel to y sides defines one channel and its walls. Then, adding adjacent copies of the ensemble forms the capillaries for structure A, B or C.

This process results in a series of parallel capillaries. Each capillary is a periodic repetition in the longitudinal *x* direction of an elementary pattern shown Figure 4 left panel. The blue region in the rectangle is the melted metal of a height 100 μm while the white region is an empty cavity bounded by the walls of the blue region. The cavity constitutes the elementary cell of the capillary. Note, that the parallel capillaries are separated by the walls of a width at least larger than *W*3. Thus, water can only flood from one capillary to the other by passing over the wall. The geometric characteristics of the fin shape is given in the table of Figure 4 right panel. In the elementary cell, we distinguish the straight channel, called *channel*, from the wider region constituted by the fins, called *V* region. Figure 2 right panel shows the top view of the CAD drawing of the three sets of capillaries called structures A, B and C. Their respective widths are 5, 5 and 6 mm. Diameter and height of the platform are 50 and 10 mm respectively. Left panel of Figure 2 is a photo of the round platform and three adjacent structures manufactured by SLM at the center.

Geometric specification is not sufficient to determine the functionality of the structure to promote directional wicking since initial surface properties and powder grain size influence wetting dynamics of each structure. A slight initial platform roughness is necessary to enhance powder adhesion. Here, the initial platform roughness was 207 RMS. However, increasing roughness may block the wetting front. Another influential parameter is the surface roughness of the laser sintered part. On one hand, we used steel powder with a grain diameter of about 5 μm. On another hand, the resolution of the final product, it is also strongly influenced by the laser beam focus. Here the final resolution was 30 μm, and it can be seen on Figure 1 and Figure 3 that the channel walls deviate slightly from the isometric CAD drawing of Figure 1 (middle) and exhibit small scale defects. In addition, the steel pattern surface (Figure 1 (left)) is rough compared to the resin surface (Figure 1 (right)) of [16].

We thus obtained three manufactured structures A, B and C positioned side by side on the same steel plate. Each structure is wide enough for a drop to be placed on it without touching its boundaries. A picture and a schematic of the resulting device are shown Figure 2.

### 3.2. Experiment

The spontaneous drop motion on A, B and C is captured by videos of three liquid drops successively placed on these structures. For this purpose, we used a Keyence VHX-600 microscope equipped with a VH-Z20R and VH-Z100R lens. The platform was placed horizontally under microscope during the experiment. In addition, photos and videos were captured by Samsung Galaxy Note 8 and Olympus Stylus SH-50. In all the experiments the distilled water has a contact angle of about 50 degrees.

**Figure 5:**
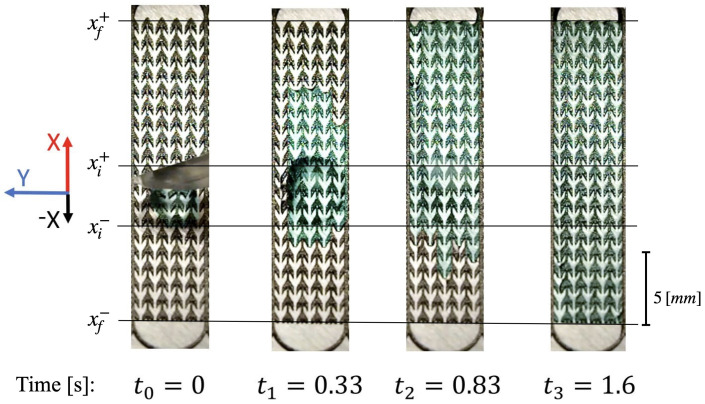
Time evolution of directional drop wicking on structure B. The bottom of the channel appears as white and the top of the structure is dark. Water is colored for better visibility. The first picture at time $t_{0}$ present the pipette that places a water drop on B structure. At time $t_{1}$ the drop has begun to wick through the channels. At time $t_{2}$, the structure is completely flooded in the positive direction while wicking progress is slower in the negative direction. Finally at time $t_{3}$ transport in the negative direction is completed.

Qualitatively, the wicking dynamics is similar on all structures. Especially, wicking starts as soon as the drop is placed on it at initial time $t_{0}$. Figure 5 displays four snapshots representing the different steps $\left(t_{0},t_{1},t_{2},t_{3}\right)$ of the evolution of a single drop placed in the middle of structure B. The snapshots were extracted from the video. The video can be found in the experiment subsection of Supplementary Materials. At the beginning, the water fills only the capillaries where the drop is initially setting, i.e., the three center capillaries. The mostleft and mostright capillaries are progressively flooded in a second step.

The wicking is faster in the +x than in the −x direction. The mean velocity of wicking in each direction is estimated by recording the initial positions of the front ($x_{i}^{+}$) and the back ($x_{i}^{-}$) of the drop at $t_{0}$ and by measuring the time taken to reach the ends ($x_{f}^{\pm }$) of the structure (Figure 5). With these notations, the average velocity in each direction is

(1) $$\langle V^{\pm }\rangle =\frac{x_{f}^{\pm }-x_{i}^{\pm }}{t_{f}^{\pm }-t_{0}}.$$

**Figure 6:**
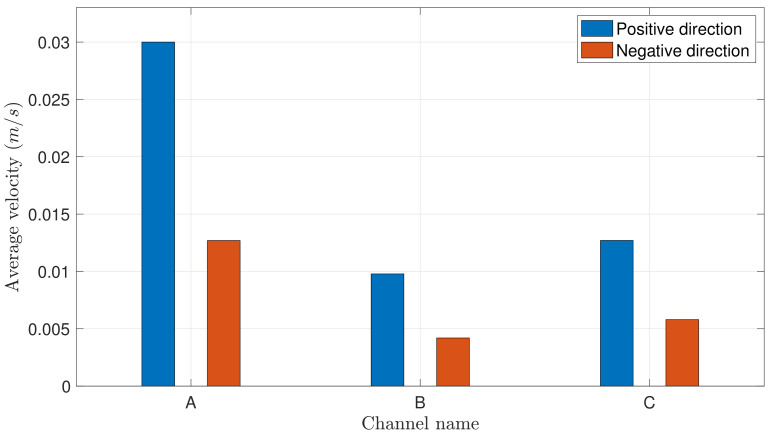
Average velocity absolute values of water wicking in *x* and −*x* directions for the three different channels A, B and C.

Figure 6 documents absolute values observed in these two directions on the three structures A, B, and C. On each of these three structures the average velocity in the negative direction is nearly half the value observed in the positive direction. According to the capillary sizes shown in the table of Figure 4, we observe larger velocities in both directions *x* and −*x* on thinner structures.

To better understand, the wicking dynamics in both directions, in the following paragraph, snapshots of the front evolution at the cell scale are displayed.

**Figure 7:**
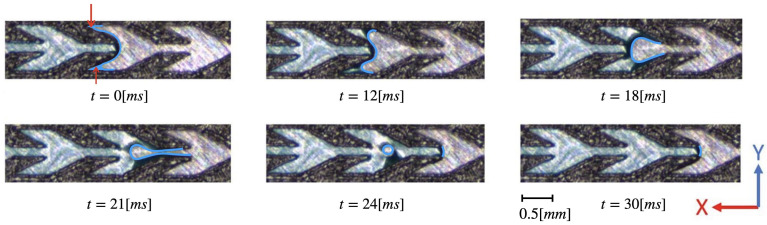
Evolution of water transport in channel type *B* in the negative direction. Experimental conditions are the same as in Figure 5. Water is colored in blue for better visibility and the triple line is highlighted by a bold blue line. Both red arrows for the panel at t=0 [ms] indicates the presence of water in the tip of the fin.

In the negative direction, we were able to capture slow down of the front dynamics during the experiments of Figure 5. In the first snapshot (t=0 [ms]) of Figure 7 the front is blocked at the right (−*x* direction) of the first cell in the thin straight channel. However, the second cell is not completely dry: a small amount of water can be observed in the tip of the fin (see red arrows in the first panel of Figure 7). The fin volume is progressively filled up by water, first slowly till t=12 [ms] and then faster until t=24 [ms]. At t=30 [ms] the water continues into the next cell in a same way as for the snapshot at t=0 [ms]. Therefore, the front remains blocked at the entry of the second cell but the water finds another path to advance to the second cell. The reason of the existence of the thin water film connecting the blocked front is unknown. One possibility is that water is flowing on the upper surface of the pattern. However, the video cannot corroborate or infirm this assumption.

**Figure 8:**
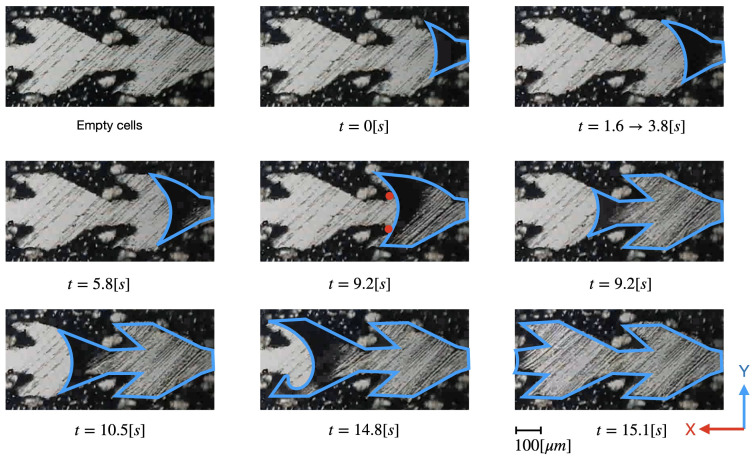
Evolution of water transport in channel type *A* in the positive direction. Experimental conditions are the same as in Figure 5. Many chemical defects slow down the dynamics. Water is delimited by a blue line for better visibility. Red dots of the snapshot at t=9.2 [ms] highlight the acute edges discussed in the text.

Due to fast progress of the liquid in positive direction, we were not able to have similar snapshots for the front evolution. However, during the first experimental tests, the front progress was much slower than reported in Figure 6. We believe that chemical impurities on the surface are responsible for pinning effect as reported in [40]. Figure 8 shows the snapshots front evolution over two cells of the fine structure A in this context. The triple line displays a concave line as described in the literature. Between the time t=1.6 [s] and 3.8 [s], the front is asymmetric and remains almost static. We argue that the chemical and topographic defects of the melted metal surfaces visible in the first panel are responsible for this pinning effect. Once the front is depinned, the curve retrieves the y↔−y parity symmetry. At t=9.2 [s] the front is near touching both acute edges highlighted with red dots. Below the camera time resolution, the front passes through the straight channel and the water floods the second cell. A similar scenario occurs for the second cell. Note that this time the front breaks the symmetry in y↔−y before it touches both acute edges however, that does not affect the sudden front burst. Therefore, there is a high velocity contrast between the flooding of the *V* region and the fast evolution by touching the acute edges.

The numerical simulation in Section 5 is performed by neglecting the surface defects but reveals an analogous front burst due to the acute edges. Therefore, the snapshots of Figure 8 can help to understand the liquid progress in a clean surface as well.

We discuss the different steps of the dynamics in Section 5, with the help of the numerical simulation that returns a much smaller time resolution.

## 4. Physical Model and Numerical Methods

The wicking dynamics is governed by the liquid properties ρ,μ,σ (density, viscosity and surface tension) and by the interaction between fluid and metal surface through the static contact angle θ. We employ two methods to numerically simulate the two-phase flow: the liquid and the gas. Most of simulations are performed using the Volume of Fluid (VOF) method [41,42,43]. The temporal evolution of the velocity field in each fluid phase is governed by the Navier-Stokes equation in viscous and incompressible fluid. At solid boundaries we consider wall adhesion. The liquid/gas interface is modelled by a region of non-zero thickness where one passes continuously from one phase of the fluid to the other. Finally, at the triple air/liquid/solid interface there is the so-called triple line. At equilibrium, the static contact angle θ characterises the wettability. Out of equilibrium, the macroscopic contact angle depends on the front dynamics and it differs from the static contact angle [44]. However, at the mesoscopic scale, it is still relevant to consider the contact angle θ [45]. Therefore, following [42] we assume that the contact angle during the front dynamics is equal to the static contact angle θ. Equations of the VOF method are detailed in the present framework in [37].

However, the main drawback of the this method is the interface diffusion which may underestimate the driving forces at the interface. Then, when the interface diffusion becomes large, we employ the color gradient Lattice Boltzmann Method (LBM). The equations of the LBM code is described in [46]. The relevance of each code depending on the simulation is discussed in Section 5. Usually, generating structured mesh for VOF would be more time consuming than for the LBM method. However, for compensation we have applied symmetry to tetrahedral mesh for 2D case to increase the computational speed and avoid asymmetric behaviors due to unstructured mesh. We have not used the symmetry for 3D simulation. Then, the 3D simulation requires more CPU time than LBM method. The type of mesh and the number of cells is summarized in Table 1.

In order to determine the main parameter of the dynamics, we estimate the following dimensionless numbers: Reynolds number (Re), Capillary number (Ca), Weber number (We) and Bond number (Bo) (Table 2).

In contrast to the quasi-static assumptions found in literature, the simulation shows that the flow velocity is about 1 [m/s] and the Reynolds number is about 100 during fast phases of the transport. Then, the flow is far from equilibrium and the inertia plays an important role. During the fast flow phase, the surface tension is in competition with the inertia force (We≃1). Otherwise, the surface dominates over the inertia force (We≪1). Moreover, since Ca≪1 and Bo≪1 the surface tension dominates over the viscous force and the gravity force. Hence, we neglect the gravity force as in [2].

Because of the computational cost, the numerical simulation is performed in a single capillary composed of 5 to 8 elementary cells repeated in the *x* direction of the geometries A, B and C. In a first step, we consider a 2D problem. The 2D simulation is obtained by considering the vertical *z* direction as the invariant direction. The most significant results are in 3D, but 2D simulation helps to understand the key role of the edges exhibited by the fin-shaped geometry.

In all simulations, we assume fixed and constant atmospheric pressure of the gas phase.

**Figure 9:**
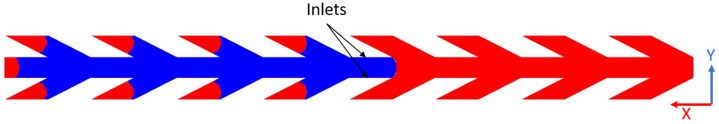
VOF two-dimensional simulation with $\theta =50^{\circ }$ and the geometry of the horizontal section of channel A.

The water is introduced in the the structure in two different manners: First one considers an initial volume of liquid and the second one considers a constant liquid flux throughout the simulation. In most 3D simulations, as for the experiment a small liquid volume is placed on the channel at the time instant t=0. The initial liquid volume is a cuboid 0.3 mm high and the same W2 width as the channel and the volume in the channel below the cuboid is also filled up. This total volume is much smaller than the droplet volume in the experience. To avoid the limited liquid volume, a liquid flux in middle of the channel at a constant rate of 1.06 mm^3^/s is imposed all along the simulation. This condition is applied to 2D simulation too. In this case, the liquid is injected at two inlets positioned in the middle of the channel boundary of one elementary cell as indicated in Figure 9 with the same flux rate.

The simulation provides a description of the wicking dynamics at smaller space and time resolution than the experiment. Moreover, simulation allows to explore a wide range of physical parameters and providing the optimal parameters that enhance the directional transport.

## 5. Water Wicking Simulation

When compared with our experimental conditions, the 2D simulation amplifies the role of the edges of the fin-shaped channel walls and retrieves (see Section 5.1) the selective pinning predicted by the Gibbs criterion near obstacles exhibiting edges [47]. In three-dimensional geometry, however, the role of the edges is mitigated by the channel bottom and the VOF results come closer to our experimental observations.

### 5.1. 2D Simulation

The 2D variant of the numerical code described in Section 4 represents a three-dimensional two-phase flow invariant in the *z* direction, between infinite walls based on the boundary in the (x,y) plane of the elementary cell defined in Figure 4. Such a flow is not expected to resemble our experiment closely, and especially its initial condition that depends on *z*. Instead, we impose a liquid flux (10.6 [mm^2^/s]) at inlets located on both parallel sides of the domain boundary as on Figure 9. These conditions are reminiscent of reference [47] that describes a (*z* dependent) liquid flooding a flat surface decorated with obstacles that exhibit wedges.

**Figure 10:**
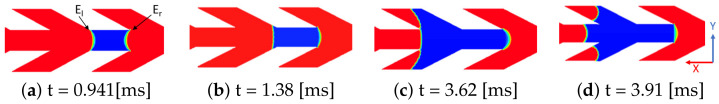
Snapshots of the asymmetric transport using the 2D VOF simulation. Parameters as in Figure 9.

In our simulation we observe that the liquid, driven by the capillary force, begins by completely filling the straight narrow channel that includes the inlet (Figure 10a). The left and right fronts are pinned at edges called $E_{r}$ and $E_{\ell }$, respectively located at the right and at the left of the inlet. To move again, a driving force is required. In our context, it is the pressure due to the continuous flow at the inlet. Since $E_{r}$ is much sharper than $E_{\ell }$, the left front rapidly depins whereas at $E_{r}$ the interface bulges gradually while the triple line remains pinned (see Figure 10a–c). The right $E_{r}$ edge is thus responsible for front pinning while the edge at $E_{\ell }$ is not: this is the key of the directional transport in 2D geometry. Note that the acute wedges at the end of the fins do not pin the front moving to the left since they are wetted by water on both sides (Figure 10c,d). References [2,28] already suggested this quasi-static explanation for the directional transport, based on contact angle hysteresis. At t=3.62 [ms] (Figure 10), the left front is similar to that of the experiment at t=9.2 [s] (Figure 8). However, the 2D simulation does not accurately describe the flow in the fins, as an air volume is trapped in the fins, blocking water progression (see Figure 10d), a phenomenon that we do not observe either in the experiment or in the 3D simulation.

### 5.2. 3D Simulation at 50° Contact Angle

**Figure 11:**
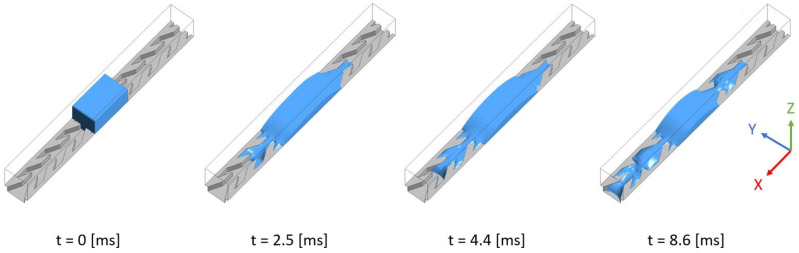
3D simulation of liquid wicking driven by capillary forces on channel A. Blue color represents liquid volume. Contact angle is 50°. The simulation domain is eight elementary cells long. The parallelepiped size is $\ell_{x}=0.7$ mm, $\ell_{y}=0.38$ mm, and $\ell_{z}=0.4$ mm.

In the 3D simulations the geometry of one cell is identical to that used in the experiment. However, one focuses on a unique series of lined up cells and periodic boundary conditions are imposed in the lateral *y* direction. An initial condition similar to the experiment consists of a rectangular ‘droplet’ placed on the structure (Figure 11). The case of water introduced by an inlet at the bottom is studied at the end of Section 5.

In these two cases, the 3D simulated flow remains symmetric regarding the y↔−y in contrast to the experiment (Figure 8). This corroborates the hypothesis made in the Section 3 that asymmetry comes from local defects and not from a spontaneous symmetry-breaking of the flow. Moreover, 3D simulation mimics the intermittent behavior suggested by the videos at the end of Section 3, alternating fast and quasi-static steps. The smaller resolution of the numerical approach uncovers the details of these different phases which we now describe in the case of a 50° contact angle.

Just after having initially been placed on the structure (at t=0) the liquid parallelepiped deforms quickly to a smoother shape and begins to wick on the channel bottom in the two directions *x* and −x. This can be seen on Figure 11 that shows four snapshots of the simulated liquid volume: at time t=2.5 [ms] it has flooded one cell to the right of its initial position, and two cells to the left. However, the right front remains pinned until t=4.4 [ms] while the left front floods one cell further and proceeds in the *x* direction. After t=4.4 [ms] the right front floods one cell more to the right before stopping. Meanwhile, the left front continues proceeding to the left until it reaches, at t=8.6 [ms], the end of the computational domain.

**Figure 12:**
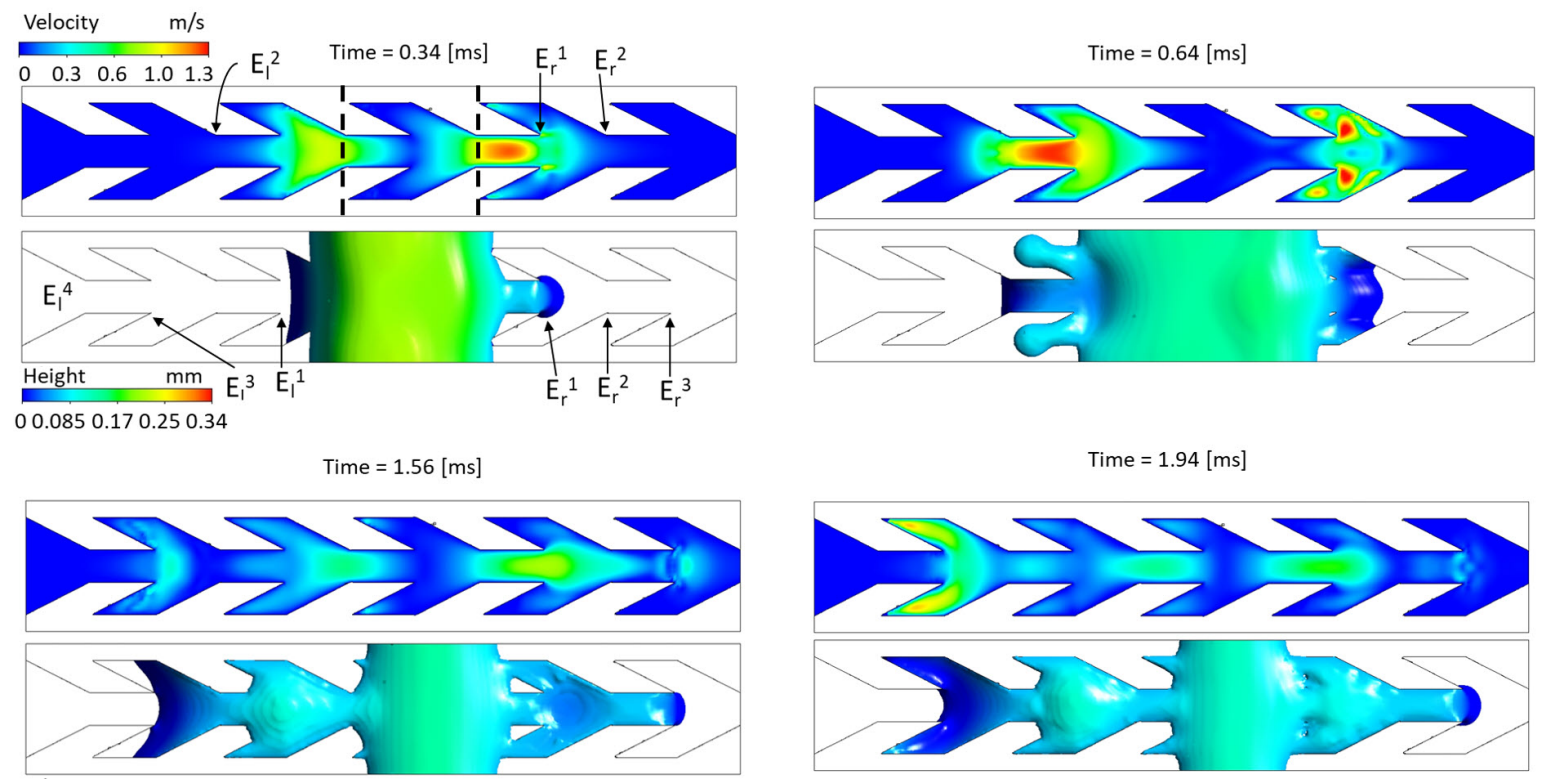
Simulated liquid velocity and free surface height during the wicking of a parallelepiped of liquid over a single fin shaped channel. For each sub-figure, the color scale displays the norm of the velocity field in the upper panel and the liquid height distribution for the bottom panel. Contact angle is 50°. The simulation domain consists of 6 elementary cells. The initial liquid parallelepiped is one elementary cell long. Figure credit from [37] Figure 4.

Figure 12 investigates the details of the above described pinning and fin filling processes in the −*x* and *x* directions. Figure 12 shows snapshots of a simulation started from an initial parallelepiped shorter than in Figure 11, and slightly longer than the elementary cell. The figure is divided into four panels captured at different times. Each panel documents liquid height below, and the velocity field at z=0.05 [mm] above. Since the two-dimensional approach and the discussion of Figure 11 pointed out the crucial role of convex edges, symbols $E_{r}^{i}$ and $E_{l}^{i}$ (i=1,2,…) mark the channel cross-sections that include such elements to the right and to the left of the initial liquid volume. At t=0.34 [ms], shortly after the initial condition, the first cell is filled and the water spreads to the left and to the right. The view from above of the front evolution (especially the concave left front) is reminiscent of the two-dimensional simulation sketched on Figure 10. However, this time the convex front at the right stays pinned on $E_{r}^{1}$ for a finite time after which the liquid spreads out further to the right into the wider *V* part of the channel (Figure 12 at t=0.64 [ms]). Two main reasons explain this discrepancy with Section 5.1. The first one is that a part of the triple line now lies on the channel bottom while contact angle hysteresis only occurs at the edges. Advancing by spreading on the bottom surface and circumventing edges is easier, and it is what we observe at t=0.64 [ms]. The second reason is the velocity field at t=0.34 [ms] which suggests that inertia should not be neglected, differently from the quasi-static approach of [16].

Once the fins between $E_{r}^{1}$ and $E_{r}^{2}$ are filled (shortly after t=0.64 [ms]) the right front continues to advance in the straight narrow channel. At t=1.56 [ms] it is at $E_{r}^{3}$. However, this time the flow velocity is about two times slower. Therefore, the right front spreads a little beyond $E_{r}^{3}$ before immediately receding (t=1.94 [ms] in Figure 12). Surface tension causes the back flow by decreasing the free surface, and thereafter the front remains pinned. Kinetic energy is now dissipated.

On the opposite side, the left front approaches $E_{l}^{2}$ at t=0.64 [ms]. When it touches the acute wedge $E_{l}^{3}$ at t=1.56 [ms], the flow suddenly accelerates as observed for the experiment (Figure 8). At the time instant t=1.94 [ms] the velocity is about 1 [ms] in the fin volume. The main explanation of this acceleration is that the tapered geometry of the fin increases the ratio of the wet solid surface to the liquid volume in the fin implying the increase of the ratio of the driving capillary force over the inertia force. During the fin flooding, the kinetic energy is only partly dissipated when the liquid completely wets the fin surface. This results in a reverse flow in the fins, converging to the middle of the channel between $E_{l}^{2}$ and $E_{l}^{3}$ (Figure 12 at t=1.94 [ms]). This back flow increases the free surface area of the liquid. Thus a fraction of the kinetic energy is transformed into surface energy. This potential energy is added to the capillary effect due to the straight channel between $E_{l}^{3}$ and $E_{l}^{4}$. A fast flooding then occurs in this channel.

**Figure 13:**
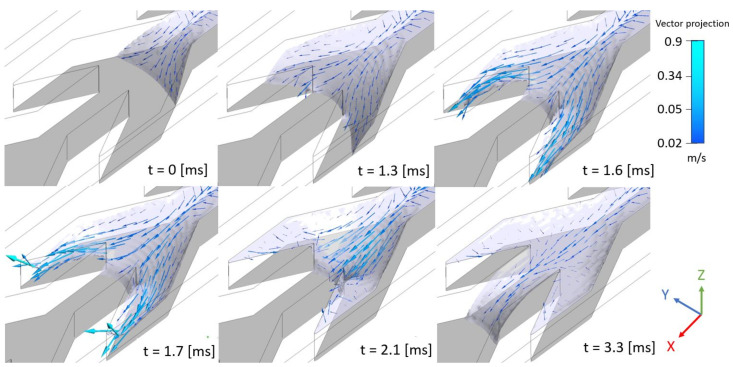
VOF simulation detailing fin filling in the *x* direction. The continuous color represents the liquid free surface. Arrows display the velocity on the liquid free surface with a color code for the amplitude. However, instead of being started from an initial condition as for Figure 11 and Figure 12, the simulation is issued from the continuous fluid inlet condition described in Section 5.4. The time is set to zero when the liquid enters the *V* region.

The same trend is observed on Figure 13 that details the fin filling process and represents the free surface and its velocity from a simulation in which the water input was different, however the figure specifies the behavior of the front near a cross section that it reaches at t=1.3 [ms], and plays the role of $E_{l}^{3}$. Observe the velocity increase that develops in the fins before returning to the median region. Meanwhile the front advances slightly in the straight channel before receding during a pause that lasts over 0.8 [ms]. However, after this pause at t=2.1 [ms] the velocity field is non-zero in the median region. The relaxation of the free surface is responsible for this significant velocity field. Then, this kinetic energy helps the front to flood the next straight channel to the left. A video presenting the evolution presented here can be found in the simulation subsection of Supplementary Materials in the experimental part.

Numerical simulation provides a better understanding of the dynamics during the fast phase that video of the experiment cannot capture. In particular, the interplay between inertia and surface tension explains that the dynamics remains fast after the filling of the fin region.

The simulation differs from experiment during the slower phase when the front advances in the wider part of *V*. The simulated front dynamics is faster, regular and symmetric (y↔−y) contrary to the stick-slip motion and to the asymmetric of the shape front described in Section 3. This proves the role of surface defects which are responsible for the pinning effect that slows significantly down the dynamics. In addition, in the simulation the left directional transport is limited by the amount of liquid deposited and we expect that the meniscus will stop before reaching the end of the channel in a longer device with the initial condition considered here. Likewise, the simulated meniscus on the right of the fluid volume is pinned at $E_{r}^{3}$ whereas in the experiments it continues unevenly to the right. A possible cause is the initial amount of liquid, which is about 50 mm^3^ in the experiment whereas it is only 0.1 mm^3^ in the simulation. An attempt to circumvent the problem of initial volume is studied in Section 5.4.

The number of cells filled by the water also depends on the static contact angle between liquid and the structure and on pattern size A, B or C. The next section investigates the influence of the contact angle and the pattern size.

### 5.3. Influence of the Contact Angle and Geometry

In the first part, only the static contact angle θ varies but all other parameters remain the same and the pattern size is that of structure A. In other words, we simulate a change of substrate wettability.

We observe that increasing the static contact angle θ decreases the mean front velocity in the positive direction *x* and the right front remains pinned. This behaviour can be explained by the decrease of capillary forces as the contact angle increases. Beyond $\theta_{lim}≃70^{\circ }$, the wicking through the capillaries does no longer occur. The rectangular water volume converges rapidly to an equilibrium state which is similar to the steady-ridge on a flat homogeneous substrate described in [40] and with periodic boundary conditions in *y* direction.

**Figure 14:**
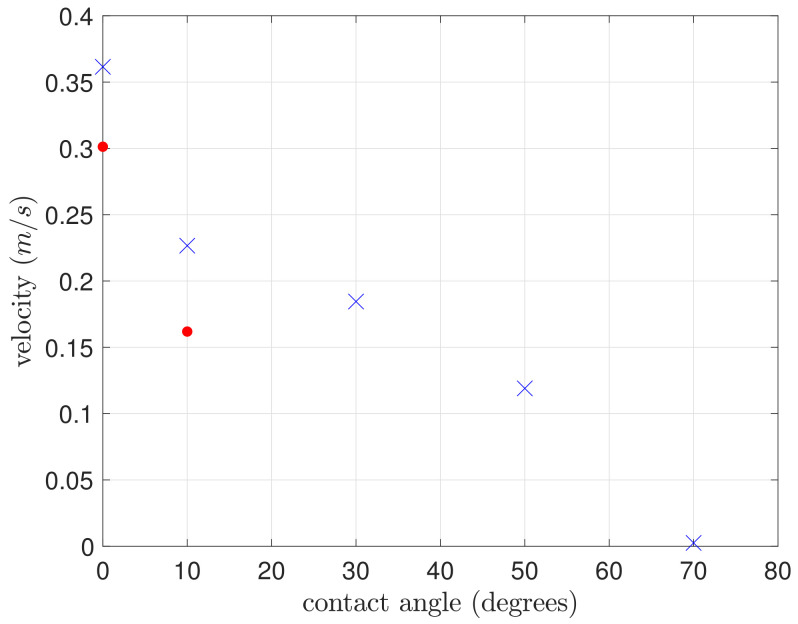
Average velocity of the front as a function of the static contact angle θ. Blue crosses indicate front velocity in the +*x* direction while red dots indicate the front velocity −*x*. Beyond $\theta \geq 30^{\circ }$ the front in the −*x* direction is pinned and then velocity is not indicated. The geometry corresponds to the smallest pattern A of Figure 4.

**Figure 15:**
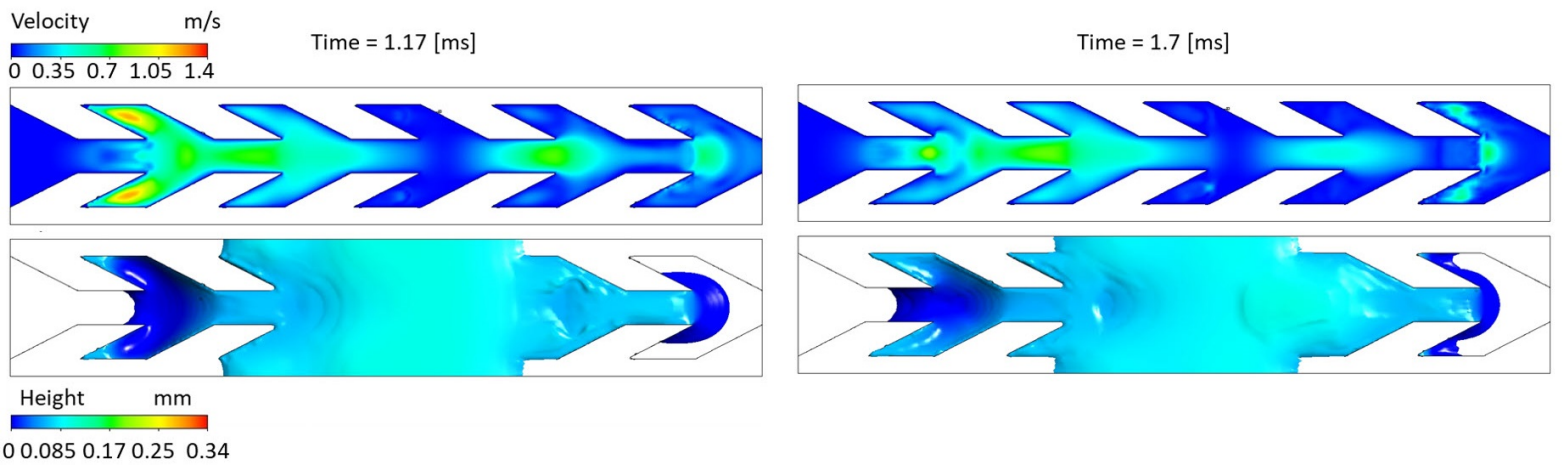
Spreading of water beyond the pinning point (t=1.17 ms) and backflow (t=1.70 ms) for a static contact angle $\theta =30^{\circ }$. Velocities and liquid height are documented at the top and bottom of each figure, respectively. The initial condition is a two elementary cells long liquid parallelepiped. The channel geometry corresponds to the pattern A.

Conversely, decreasing θ from $50^{\circ }$ speeds up the wicking front progression in the *x* direction (Figure 14). However, this affects the front pinning at $E_{r}^{3}$ in the −x direction. For instance, at $\theta =30^{\circ }$, the fluid spreads over a larger area before retracting and remaining pinned at $E_{r}^{3}$ (Figure 15). This dynamics results from a competition between inertia and surface tension. There is a critical value, noted $\theta_{c}<30^{\circ }$, below which the front is no longer pinned at $E_{r}^{3}$ and water spreads in both directions. Even in this case the wicking is slower in the −x than in the *x* direction. Though the front is no longer pinned, the $E_{r}^{3}$ edges constrain the flow to spread out in a thin film beyond $E_{r}^{3}$. Since viscous dissipation is strongly related to the height of liquid films, the kinetic energy is almost totally dissipated passing through $E_{r}^{3}$ in the −*x* direction as a thin film. Thus, the flooding in the *V*-region is slow and this explains the slower wicking than in the *x* direction.

To quantitatively study the transport efficiency, we estimated the transport speed averaged on the few cells that were filled in the *x* direction (Figure 14). The mean velocity in both directions decreases as the contact angle increases. The decrease in the *x* direction is not linear. Between 0 and 10° the velocity is divided by 1.6 while in the larger range [10°, 50°] it is divided by 2. For $\theta =0^{\circ }$ or 10° the asymmetry of the wicking is weak, as indicated by the similar values of velocity in the *x* and −*x* directions. Thus, the contact angle $\theta_{c}≃30^{\circ }$ represents an optimal value of the directional wicking since it is the maximum velocity of the front in the *x* direction for which the front is still blocked in −*x* direction.

We also simulated the influence of the other geometries B and C of the experiment. The geometry does not qualitatively change the scenario described above. The three structures exhibit a similar trend for the contact angle dependency of the average velocity, showing in all cases an upper limit $\theta_{lim}$ for the wicking in the positive direction and a critical value $\theta_{c}$ below which the pinning effect vanishes in the negative direction.

**Figure 16:**
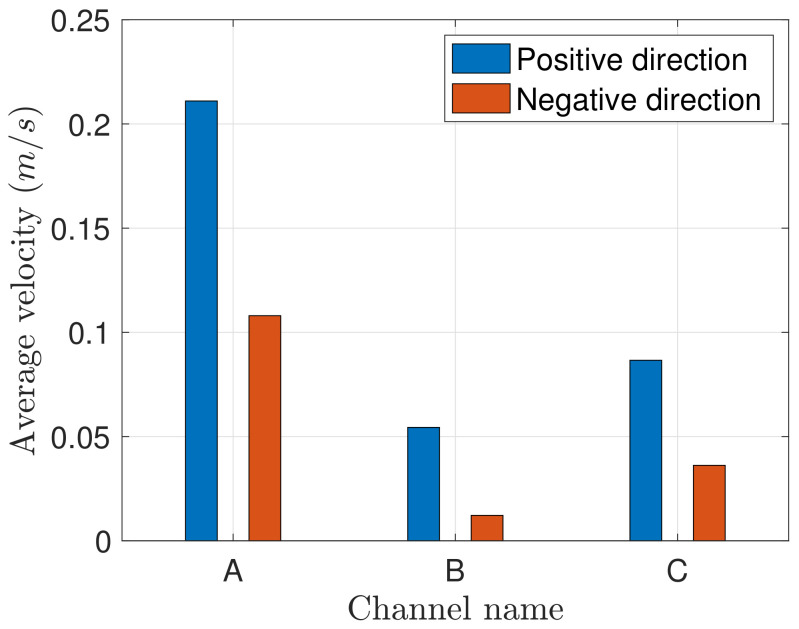
Influence of the pattern geometry on the average velocity of the front in the positive *x* direction. The contact angle is $\theta =30^{\circ }$.

The velocity of fluid wicking is influenced by pattern size (Figure 16). This is especially visible on structures A and C, for which the velocity difference is maximum for $\theta =30^{\circ }$. This result is qualitatively very similar to the experiment (see Figure 6). Indeed the capillary force over the inertia ratio is larger in smaller cells: dividing the sizes in the (x,y) plane by factor *f* divides the water mass by $f^{2}$ whereas the capillary force, proportional to the contact line length, is divided by *f*.

Despite the qualitative agreement with experiment, the average velocity of the liquid spreading through the capillary is about ten times that of the experiment. Surface defects as shown in Figure 3 are known to slow down the wetting dynamics [40]. Even considering a smooth surface, the low volume of water in the simulation compared to the experiment is another possible reason for this discrepancy. Firstly, the low volume does not make it possible to average out over a large number of cells implying a large uncertainty. Secondly the Laplace pressure in a small droplet is higher than in a larger one because the Laplace pressure is proportional to curvature. In the next section, we propose a continuous flow to explore the dynamics without the constraint of a limited volume.

### 5.4. Continuous Flow

**Figure 17:**
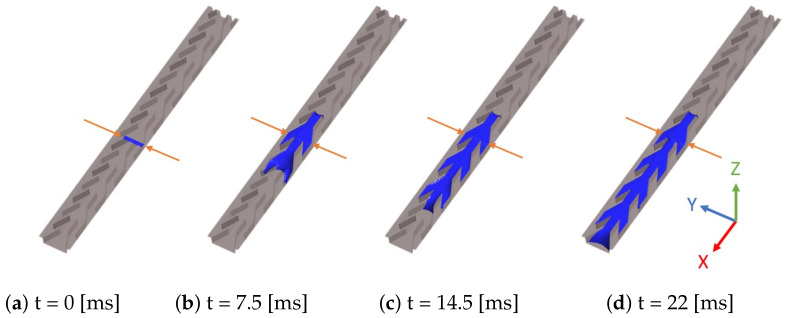
VOF simulation with an inlet at the bottom of channel A. Inlet flux is 1.06 mm^3^/s and $\theta =50^{\circ }$,

To circumvent the problem due to the low volume of liquid in the simulation, we impose a continuous inlet condition at the bottom of the straight channel in a similar way to the 2D simulation. An interesting feature is that this boundary condition is reminiscent of the context of the bipolar plates of a fuel cell as the reaction at the cathode produces a continuous water flow. We simulated the water production by constant flux (1.06 [mm^3^/s]) through a rectangular surface positioned at the bottom of the channel (Figure 17).

The simulated dynamics displays an uneven wicking progression in the positive direction as described in the previous sections. This time, the spread in the positive direction is no longer limited. In contrast, the front is still definitively pinned in the −*x* direction at the $E_{r}^{3}$ edge. That proves out that the cell geometry is effective in obtaining a directional wicking.

**Figure 18:**
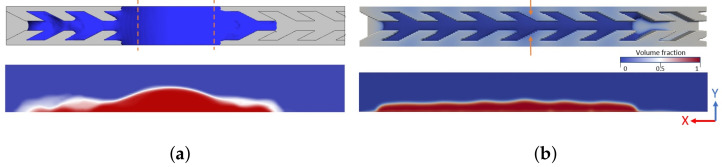
(**a**) Diffusion at the water/air interface in VOF simulation with compressive scheme. Parameters are as in Figure 11. (**b**) LBM simulation performs clear phase separation without excess diffusion at the interface with an equal number of cell elements. Red arrows indicate the location of the inlet. Contact angle $\theta =50^{\circ }$.

**Figure 19:**
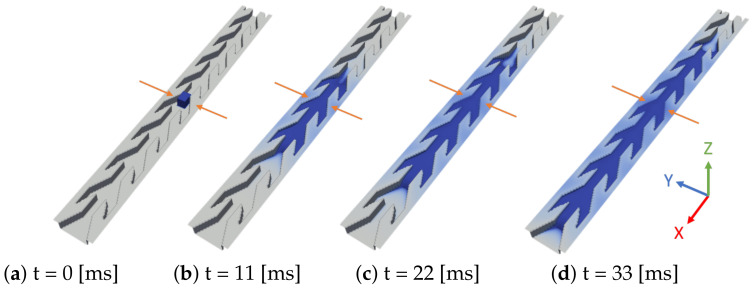
LBM simulation with an inlet (red arrows) at the bottom of channel A. Contact angle $\theta =50^{\circ }$.

Nevertheless, the long duration of VOF simulation increases the numerical interface diffusion. Figure 18a displays the large diffusion of the interface between water and air. The overestimate transition region at the water-air interface impacts the modeling of the driving Laplace force and the capillary force at the triple line. To decrease this numerical effect, we used Compressive Interface Capturing Scheme for Arbitrary Meshes (CICSAM) that proved to be a better choice than the Geometric Reconstruction Scheme (GRS) or High Resolution Interface Capturing (HRIC). To reduce diffusion at the water/air interface local mesh refinement can be used which adds to the total computation time by mesh generating for each specified timestep. Indeed, the main drawback of the VOF simulation is that it exagerates the numerical interface diffusion [48]. We believe that the interface diffusion may underestimate the capillary force and then mislead the wicking dynamics simulation. The color gradient Lattice Boltzmann Method (LBM) used in [46] provides an alternative to model interface dynamics. The interface diffusion is drastically reduced by LBM simulation compared to VOF for a similar mesh refinement (Figure 18). Using similar inlet conditions, the LBM simulation mimics the above described fast/slow alternating steps without definitively pinning the right front: the latter stops at the entrance of the fins but continues its progression after some time (Figure 19). The steps of the front progression in the negative direction (Figure 7) are retrieved in the LBM simulation. Even if the front is pinned at the sharp corner $E_{r}^{i}$, a small amount of water in the tip of the fin is present. The volume in this region grows and progressively fills the volume of fins. Then, the water continues to the next channel. Depinning does not take place but the water bypasses the $E_{r}^{i}$ edges. The details of the LBM simulation suggest that a thin film develops on the surface of the structure and opens a path to the fin. Nevertheless, according to [49], it is possibly an artifact of the LBM simulation due to instability at the interface between the liquid and gas phases.

However, the similarity with the experiment leads us to infer that such thin liquid films actually exist. An explanation for these thin films could be the presence of surface irregularities at the microscale made by SLM manufacturing (Figure 3). The interstices between defects act as capillaries into which water may infiltrate and bypass the corner $E_{r}^{i}$. Therefore, the defects decrease the dissymmetry of water wicking by, on one hand, allowing a wicking in the negative direction and another hand, by slowing down the front (stick-slip motion) in the positive direction.

Finally, according to the VOF and LBM simulations the wicking in the negative direction occurs only if the water finds a path over the structure or a path due to the defects. Thus, the depth of the structure should adapted to the liquid volume to transport in order to avoid overflooding above the structures.

## 6. Conclusions

Motivated by the problem of water drainage in a fuel cell, our study demonstrated the feasibility of the directional spreading of a water drop on a structured steel substrate. The fin geometry of the micro-channels was based on literature, in particular [16]. However, we employed a conductive substrate in contrast to the non-conductive PDMS polymer of [16] and to organic substrates [5,7,8,10,39]. Because of the low wettability of water on metal, there was no evidence that the devices reported in the literature could apply in the current framework. Moreover, the manufacturing of channels with complex small-scale geometry was a challenging problem. To our knowledge, it is the first time that this type of structure has been manufactured additively with a resolution down to about 30 μm. Experiments using three different pattern sizes provide a wicking in a preferential direction. Its efficiency is correlated to the structure scale and this highlights the importance of making small structures.

For an in-depth understanding of the mechanisms involved in the liquid wicking we used a three-dimensional numerical simulation of multiphase Navier-Stokes equations taking into account free surface, contact angle and inertia. This approach contrasts with the quasi-static description in the literature [5,16,17,18]. We used two complementary methods: Volume of Fluids (VOF) and Lattice Boltzmann Method (LBM) to capture the complex dynamics. The VOF and LBM simulation retrieved the jerky dynamics of the wicking in the desired direction. The jerky dynamics brings into play inertia during burst phases. The fluid inertia has an influence on the onset of depinning and has therefore to be taken into account in the design of the channel shapes. This fact is reminiscent of the imbibition in porous media for which inertial forces may affect fluid front displacement dynamics as proved by [50]. In the negative direction, i.e., unwanted direction, the simulation reveals how water may bypass the edges responsible to the pining effect.

Numerical simulation provides a limit angle around $70^{\circ }$ beyond which the liquid will no longer spread through the micro-channels, whereas the optimal contact angle of the asymmetric spreading is about $30^{\circ }$. This value is significantly below metal/water values which are typically superior to $50^{\circ }$. Consequently, the parameter range for designing efficient microchannels is narrow, pointing out the key role of numerical simulation in the design process.

LBM simulation tracks the gas/liquid interface better than VOF. Nevertheless, the BGK code in LBM that we used can be unstable at realistic Reynolds numbers. Other classes of LBM deserve to be tried in the future, as proposed in [51,52] for instance. Thus, a first outlook is related to the improvement of the numerical tools for the numerical simulation. Moreover, taking into account the surface defects in the simulation is a crucial outlook since our study showed that defects impact the directional wicking. An economic way to perform this task is to replace topographic defects by wettability defects. It is known to mimic the effect of surface irregularities [40].

Even if the fin shape proves to be efficient for the directional wicking, we aim at applying the numerical simulation to perform a parametric study and an optimization of the geometry structure.

In the PEM fuel cell framework, a path for improving the efficiency of the directional wicking is the surface treatment, e.g. [53], in order to achieve a lower contact angle between steel and water closer to the optimal one. In addition, in the parallel flow field, the gas shear flow drags the liquid. It is known that in confined geometry the liquid may block the gas flow [54]. However, the interaction of shear flow and wicking has thus far not been studied. Such a study will be of crucial interest in applying the structure in the parallel flow field of PEM Fuel Cells. 

## Figures and Tables

**Table 1:** Mesh properties of the Volume of Fluid (VOF) and Lattice-Boltzmann (LBM) methods used for the simulations of Section 5.

	VOF 2D	VOF 3D	LBM 2D	LBM 3D
Mesh type	Tetrahedral	Hex dominant	Pure Hex	Pure Hex
Cells number	3904	71,899–758,198	26,180	551,368

**Table 2:** The dimensionless numbers and their magnitude. *V* characteristic velocity, W1 minimum channel width and $\ell_{c}=2.7$ mm the capillary length of water.

Dimensionless Number		Magnitude
Reynolds number	$Re=\frac{\rho VW1}{\mu }$	0–100
Capillary number	$Ca=\frac{\mu V}{\sigma }$	≪1
Weber number	$We=\frac{\rho V^{2}W1}{\sigma }$	0–1
Bond number	$Bo=\frac{W1}{\ell_{c}}$	≪1

## Data Availability

The data presented in this study are available on request from the corresponding authors.

## Supplementary Materials

The following are available online at https://www.mdpi.com/1996-1944/14/3/490/s1, Video S1: Videos of experiments and simulations.

## Abbreviations

The following abbreviations are used in this manuscript:

SLM	Selective Laser Melting
IPA	Isopropyl alcohol
CAD	Computer Aided Design
LBM	Lattice Boltzmann Method
VOF	Volume of Fluid
BGK	Bhatnagar-Gross-Krook
PEMFC	Proton-exchange membrane fuel cells
